# Klamath Tribal Response to the Pandemic of COVID-19 Among Klamath Tribal Community in Oregon, USA

**DOI:** 10.1177/21649561211034470

**Published:** 2021-07-15

**Authors:** Obinna Oleribe, Rachel Miller, Misty Wadzeck, Nikowa Mendez, Joseph Tibay, Timothy Langford, Anjanette Devine, Simon D Taylor-Robinson

**Affiliations:** 1 Klamath Tribal Health & Family Services, Klamath Falls, Oregon; 2 Department of Surgery and Cancer, Imperial College London, London, UK

**Keywords:** COVID-19, community measures, social distancing, Klamath Tribes

## Abstract

**Introduction:**

Socially-disadvantaged populations are more at risk of contracting COVID-19 than those with access to better medical facilities. We looked at responses of Klamath Tribes in Oregon, USA to mitigate spread of COVID-19 in a community with a higher incidence of obesity, diabetes and coronary heart disease, compared to the general US population. This study reports on Klamath Tribes response to COVID-19 March -September 2020.

**Methods:**

Klamath Tribes Tribal Health and Family Services established a COVID-19 Incident Management Team (IMT), instituting creative programs including a Walk-In Testing Center, implementing strict infection control protocols and regular sharing of information on the pandemic and prevalence of COVID-19 amongst Klamath Tribes. All COVID-19 tests were documented with positive cases isolated and people with high risk exposures quarantined and provided with wrap-around medical and social services until recovered or past quarantine time period.

**Results:**

A total of 888 (12%) tribal members were tested for COVID1-19 between March to September 2020; 50 were found positive for COVID-19, giving a test positivity rate of 5.6% (Male – 6.3%; Female – 5.2%). No deaths have been reported amongst the local Klamath Tribes and other American Indians/Alaska Native (AI/AN) population served by the tribe.

**Conclusion:**

Despite the fact that structural inequities including income disparities have shaped racial and ethnic impact of epidemics around the world, the timely response, establishment of partnerships and proactive control of the epidemic resulted in minimal impact among the Klamath Tribal and other AI/AN populations served by the tribal facilities.

## Introduction

Since the onset in December 2019 of the COVID 19 pandemic in China, which started as a cluster of pneumonia cases of unknown cause that would later be identified as severe acute respiratory syndrome coronavirus 2 (SARS-CoV-2), the United States of America (USA) has been disproportionately affected.^1^ Although the USA has a case fatality rate of only 2.9%, at the time of writing, the USA accounts for 21.3% of globally-confirmed cases and 20.4% of the global COVID-19 mortality with 6,960,152 of the 32,730,945 of confirmed cases, and 202,478 out of 991,224 of all COVID-19 deaths worldwide being US citizens.^2^

In the USA, COVID-19 does not affect everyone equally. It has exposed inequities in the healthcare system, with those socially disadvantaged being less able to seek adequate medical help.^3^ In part, the disproportionate impact of the COVID-19 pandemic on some communities is a result of structural factors that prevent effective social distancing.^3^ Populations with underlying health conditions and who live in areas with poorer healthcare access may be most affected by COVID-19, resulting in higher mortalities. The pandemic has shone a spotlight on social determinates of health (SDH) and disparities therein. The most pervasive disparities are observed among African American and Latino individuals, and where data exist, American Indian/Alaska Native (AI/AN), and Pacific Islander populations.^4,5^ The SDH affecting this epidemic within the American Indians/Alaska Natives (AI/AN) population include poverty, low educational status, crowded living conditions, household air pollution, and lack of running water. Therefore, there has been inadequate hand-washing, poor access to healthcare due to chronic underfunding of the healthcare system, a lack of transport, and limited access to healthy foods.^6,7^

This disparity was shown in the 1918 Spanish influenza pandemic, where AI/AN communities sustained an infection rate of 24% and the highest death rate of any racial‐ethnic group, resulting in a 2% population loss.^8^ Similarly, the Sin Nombre hantavarus epidemic of the 1990s, of which about half of the initially affected population were AI/AN,^9^ and 2009 H1N1 Influenza A pandemic in which the AI/AN population experienced a mortality rate that was four times higher than in all other racial and ethnic groups combined within the United States validated this disparity.^10^

The AI/AN are unique ethnic grouping in the USA for having made peace agreements with the US Federal Government, the majority dating back to the 19th Century, which allowed Tribal groupings to be considered sovereign nations, subject to their own laws, often in exchange for land.^11^ However, this has led to many AI/AN inhabiting rural and relatively poor parts of the country that were not subject to the economic booms of recent times. The Klamath Tribes are unusual in that individual economic advancement is encouraged, rather than having a communal attitude to wealth which can lead to social disadvantage amongst some AI/AN. The Klamath Tribes have thus been one of the economically wealthier of the AI/AN and in a position to capitalise on such development for the common good.^12^

The Indian Health Service (IHS) is a US Federal Government system that provides direct medical services and public health measures to AI/AN peoples. Thus, the IHS is the main health care provider for most of the AI/AN across 36 US states.^13^ The IHS comprises 26 hospitals and more than 90 health centres and health stations. However, owing to tribal autonomy, many tribes operate their own health systems independent of IHS.^14^ While the system is essentially socialised and free at the point of entry, like the British National Health Service (NHS), it has suffered from chronic underfunding.^13^

The Indian Health Service (IHS) has reported age‐adjusted death rates for AI/AN adults which exceed that of the general US population by almost 40%.^6,15^ This may be as a result of prevalence rates of obesity, diabetes mellitus, and cardiovascular disease among AI/AN tribes that are higher than the general US population.^15–18^ Of note, these medical problems are also risk factors for greater COVID‐19‐related morbidity and mortality.^15–18^

To contain COVID-19 epidemic, the Klamath Tribes, like other AI/AN groups, took appropriate leadership steps to promote personal hygiene, social distancing, quarantine and isolation housing, and culturally appropriate healthcare preparedness.^6^ Although, at the time of writing, there were no approved prophylactic medications for COVID‐19, innovative steps including the increased telehealth in rural AI/AN communities helped to protect susceptible people by reducing or eliminating social mixing in otherwise congregate tribal communities.^19^

Although by comparison with the nine recognised AI/AN tribal groupings in Oregon, the Klamath Tribes have a relatively unique position on individual wealth that enhances individual economic development, the Klamath Tribes, along with many tribal populations, practice an integrative medical approach to life, combining mind, body and soul, rather than the traditional western medical model of reacting solely to the disease process itself. Medical intervention is performed, in full balance with societal and spiritual beliefs.

We document the initial and early-stage measures taken by the Klamath Tribes in Oregon in the face of the COVID-19 epidemic, in order to improve preparedness for future pandemics.

## Methods

As the tribe is a congregate one, decisions that have an impact on the community are made collectively. In order to assess the prevalence of COVID-19 in the tribal population and to manage its impact in the face of the pandemic, the Klamath Tribal Health & Family Services (KTH&FS) at the Wellness Center located in Chiloquin, Oregon, (USA) activated an Emergency Preparedness Incident Management Team (IMT) which managed a swift response to the outbreak, documented here from March 2020 to September 2020. The IMT instituted creative programs that ensured the protection of the tribal people including a Drive-up Testing Center at the Wellness Center.

Medical staff were trained in COVID-19 testing, two ABBOTT ID-NOW COVID-19 rapid testing machines were procured, and testing of tribal members was completed. Strict infection control processes and procedures were instituted to ensure the safety of staff and the tribal community.

Case positivity was documented on a daily basis. Results on incidence were entered into a SPSS spreadsheet (IBM Inc., Armonk, New York, USA) for calculation of statistics, both for the Community and for preparation of this manuscript.

## Ethics

Ethical approval for this study was obtained from a review committee of the Klamath Tribal Council and the Klamath County Health Board. Prior informed consent was obtained from all tribal members involved. The study complied with the precepts set out in the 1975 Declaration of Helsinki. Analysis and publication of these data were approved by the Tribal Council.

## Results

While not unique in their content, the protocols instituted by the Klamath Tribes were rapidly agreed in March 2020 at the very outset of the pandemic, through the use of videoconferencing instead of the usual congregate meeting structure. This allowed protocols and procedures to be agreed through consensus and implemented rapidly.

Infrastructure changes and facilities improvements were rapidly initiated in the clinic to provide for improved infection control, including transitioning of all exam rooms and dental procedure areas to negative air pressure rooms in the IHS facility, as well as installing touchless faucets.

Initial anti-COVID-19 mitigation techniques included mass education to high risk populations. This included provider telephone calls, provided by registered nurses (RN), medical doctors (MD), family nurse practitioners (FNP) and other healthcare professionals to the tribal elders and individuals with high risk status for COVID-19 complications. Each person was contacted individually to discuss their risk status, as well as to advise them of the need to stay at home, safety precautions to protect themselves, infection control measures to follow and how to safely access necessary supplies. High risks employees were also provided with opportunities to work remotely to reduce their individual risk; and if they could not work remotely, they were provided with paid administrative leave to support isolation.

These infection control processes included designating a specific provider team to treat potential COVID-19 patients, including a medical provider (either FNP or MD) and a registered nurse, as well as an extensive screening process. All patients with symptoms suggestive of COVID-19 were deferred from the primary entrance of the medical clinic to an alternate COVID-19 entrance for car-side testing to minimize staff and non-COVID patient potential exposures. Car-side testing was then performed. Regardless if the test result was positive or negative, all patients with COVID-like symptoms remained in the care of the COVID care team. If a patient required more thorough assessment or intervention, a designated negative pressure room was utilized for care of the patient. The clinic was split into designated care areas, with one hallway designated for potential COVID patients and another for non-COVID patients to minimize potential exposures.

Also, the IMT adopted the US Centers for Disease Control and Prevention (CDC) guidelines for COVID-19 prevention as brochures, emails and social media information was produced to regularly share information on the epidemic and prevalence of COVID-19 amongst the Klamath Tribes. All COVID-19 tests were documented, and positive and high risk exposure cases were isolated and quarantined. Direct county and state reporting was initiated. Patients were also provided with wrap-around medical and social services until they were able to meet CDC criteria for discharge from quarantine/isolation. These wrap-around services included contact-free pharmacy delivery, and delivery of all essentials for living, such as cleaning/hygiene supplies and grocery needs. These services were provided without charge to patients. Wrap-around services were then expanded to provide temporary housing for unhoused individuals who were required to quarantine or isolate, through utilization of self-contained recreational vehicle (RV) units.

The Klamath Tribe also limited travels to emergencies only, and ensured the isolation of individuals who had travelled outside the county. Whenever there were cases of COVID-19 in any tribal office, the office was closed, exposed staff quarantined, positive cases isolated and the entire office disinfected before office was reopened in line with US CDC guidelines.^20^

As the tribe is a congregate one, meeting regularly for social and cultural events, the tribe enacted policies to prevent congregate activities at burials, weddings, and traditional ceremonies.

A total of 888 tribal members were tested for COVID-19 between March to September 2020. This constituted a 12% testing rate for the Klamath Tribes (Klamath, Modoc, and Yahooskin) and other AI/AN served by our facilities, based on the total population count of 7,424 people. However, there have been no documented COVID-19 mortalities in the Klamath Tribes and serviced local AI/AN at the time of writing.

The majority of those tested were in the age group 30 – 39 yrs (19.5%) with the majority of the identified cases found in August–September 2020. Similarly, testing uptake increased progressively from March through September, 2020.

The demographics for COVID-19 infection during the period March 2020–September 2020 for Klamath County as a whole and for the Klamath Tribes are shown in Table 1. Of those tested amongst the Klamath Tribes, 50 were found to be positive for COVID-19, giving a test positivity rate (TPR) of 5.6% (Male – 6.3%; Female – 5.2%) as shown in Table 2. The highest test positivity was seen among the 0–29 yrs age group, which constituted 60% of the total positive cases with a total positivity rate (TPR) of 13.5% (0–14 yrs), 10.3% (15–19 yrs) and 8.1% (20–29 yrs); with the highest testing rate being seen in August and September 2020. The gender distribution of COVID-19 among Klamath Tribal members and other AI/AN served by our facilities mirrors that of Klamath County more widely, where there have been more females infected with percentages of 58% and 54% respectively.

**Table 1.** Comparative Demographics of Klamath Tribes and Klamath County for COVID-19 Positivity in the Period March 2020–September 2020.*

	Klamath Tribes		Klamath County	
Sex				
Female	29	58.0%	160	54.2%
Male	21	42.0%	135	45.8%
Total	50	100.0%	295	100.0%
Age group				
0–14	13	26.0%	34	11.5%
15–19	4	8.0%	13	4.4%
20–29	13	26.0%	53	18.0%
30–39	7	14.0%	50	16.9%
40–49	5	10.0%	50	16.9%
50–59	4	8.0%	45	15.3%
60–69	3	6.0%	32	10.8%
70–79	1	2.0%	14	4.7%
>80	0	0.0%	4	1.4%
Total	50	100.0%	295	100.0%
Total Tested		888		10657

*The Klamath County figures are for the county as a whole including the Klamath Tribes. Klamath County is 87% white and 4.2% AI/AN.

**Table 2.** COVID-19 Testing Results From Klamath Tribal Health & Family Services’ Wellness Center, Chiloquin (March–September, 2020).

	Positive (N) (%)		Negative (N) (%)		Total tested (%)	
Total tested	50	5.6%	838	94.4%	888	
Sex						
Female	29	5.2%	525	94.8%	554	62.4%
Male	21	6.3%	313	93.7%	334	37.6%
Total	50	5.6%	838	94.4%	888	100.0%
Age						0.0%
0–14	13	13.5%	83	86.5%	96	10.8%
15–19	4	10.3%	35	89.7%	39	4.4%
20–29	13	8.1%	148	91.9%	161	18.1%
30–39	7	4.0%	166	96.0%	173	19.5%
40–49	5	3.6%	135	96.4%	140	15.8%
50–59	4	3.4%	112	96.6%	116	13.1%
60–69	3	2.5%	117	97.5%	120	13.5%
70–79	1	3.0%	32	97.0%	33	3.7%
>80	0	0.0%	10	100.0%	10	1.1%
Total	50	5.6%	838	94.4%	888	100.0%
March	0	0.0%	11	100.0%	11	1.2%
April	0	0.0%	22	100.0%	22	2.5%
May	0	0.0%	23	100.0%	23	2.6%
June	9	6.3%	133	93.7%	142	16.0%
July	5	2.8%	172	97.2%	177	19.9%
August	10	4.8%	197	95.2%	207	23.3%
September	26	8.5%	280	91.5%	306	34.5%
Total	50	5.6%	838	94.4%	888	100.0%

## Discussion

Klamath County (Oregon) as a whole has a population that is 87% white and only 4.2% AI/AN. On March 7, 2020, Klamath County had its first case of COVID-19.^21,22^ Since then Klamath County with a population of 68,238 (2019 Census Bureau population estimate) has tested 10,657 individuals of whom 295 were COVID-19 positive.^23^ The County has also recorded three deaths to date. This gives a testing rate of 15.6%, a test positivity rate of 2.8%, and a case fatality rate (CFR) of 1.02%.^24^ This CFR is far lower than that of USA of 2.9% during the same period to September 2020. However, this rate is higher than that of the Klamath Tribes and other local AI/AN, where there have been no COVID-19 mortalities to date, despite the disproportionately higher risk factors in the AI/AN population.

The gender distribution of COVID-19 among Klamath Tribal members and other AI/AN served by our facilities mirrors that of Klamath County more widely, where there have been more females infected with percentages of 58% and 54% respectively (Table 2).^24^ This is unlike many other parts of the world, including the Nigerian experience where more males have been infected and constitute 64% of cases.^25^

While in the Klamath Tribal and other local AI/AN members served by our facilities, most of the cases have been in the population below 29 years of age, Klamath County as a whole has seen most of its cases in the population between 29–59 years of age. The low level of cases and mortality in Klamath County and among Klamath Tribal and other local AI/AN members served by the facilities could be linked to the integrative medical approach practiced by the tribe with strong community partners who joined the control process early in the epidemic. Also, the Klamath Tribe and Klamath County launched their COVID-19 Incident Management Team early. This process initiated a number of preventive activities such as drive-through testing sites that were open to the public, mobile testing clinics and hosting testing days in several communities, including Merrill, Malin, Chiloquin, Bonanza, and Crescent. To meet the needs of farmers, the County also conduct testing days on farms to ensure farmworkers had access to testing. Klamath Health Partnership and also Klamath County Emergency Management have partnered with Klamath Tribal Emergency Management to provide needed personal protective equipment and access to the KTH&FS’ Abbott ID-NOW rapid testing to first responders, healthcare workers, other essential workers, and testing for related contact tracing.

Due to home situations and/or financial limitations, many individuals have required assistance in obtaining living space in which to isolate/quarantine. KTH&FS provided residential support to individuals exposed or infected with COVID-19. Additionally, those individuals who needed assistance with food, medications, and other supplies while in isolation/quarantine, were adequately supported through KTH&FS wrap-around services. This was achieved in partnership with community-based organizations at no cost to the individuals. Klamath County Public Health, KTH&FS, Klamath Health Partnership, Central Oregon Disability Support Network, Klamath Works, Friends of the Children, and Lomakatsi Restoration Project have partnered to bring culturally and linguistically appropriate services to the AI/AN community members.

Although the COVID-19 pandemic has significantly impacted and devastated the world, our findings do not support the projection that the epidemic will disproportionately affect the Klamath Tribes and other local AI/AN served by our facilities who are part of the nation’s racial and ethnic minorities, if appropriate control measures are taken.^22^ Furthermore, despite the fact that structural inequities including income disparities have shaped racial and ethnic impact of epidemics, the timely response, establishment of partnerships, and proactive control of the epidemic resulted in minimal impact among the Klamath Tribal and other local AI/AN population served by our facilities.^26^ This also demonstrates that irrespective of the fact that people who are African American, AI/AN, or live in low-income households may be more likely to have conditions associated with increased risk of illness from COVID-19 relative to those who are white or are living in higher-income households, proper and timely public health actions can positively change the epidemic curve of an outbreak like COVID-19.^27^

The experience of the Klamath Tribes with a strong community and integrative medical approach is good evidence of the impact of good public health practice, responsible government, and compassionate medical care givers on the epidemiology of outbreaks around the world. We believe that the Klamath Tribes’ ability to combine public health measures with consensus obtained in the community through videoconferencing led to early adoption of the necessary measures in the pandemic and is an example to other communities around the world, where public consensus was not sought owing to a more didactic governmental approach.

## Conclusion/Recommendations

COVID-19 complications are disproportionately higher in individuals with comorbidities such as diabetes, heart and lung diseases. However, as the mitigation and response efforts of the Klamath Tribes have shown, despite significant population risk factors, risk does not equate a definitive effect of a pandemic. Consistent and early intervention including community-based mitigation activities, educational outreach, stringent infection control procedures, facility physical interventions, and wrap-around services are highly effective means to reduce community spread, and reduce mortality amongst high-risk populations.

Longitudinal studies to show continued efficacy of these measures should be performed to show long-term risk reduction and disease transmission prevention. Additionally, of interest would be to see how implementation of these same measures could positively benefit areas of active surge volumes of COVID-19, to see if these same measures would reduce disease transmission and mortality rates, or if these measures require implementation prior to local outbreak.

The early intervention and comprehensive community approach taken by the Klamath Tribes in management of the COVID-19 pandemic has shown that high-risk populations that have historically been disproportionately ravaged by disease outbreaks can have successful mitigation programs to pandemics if relevant steps are taken early and rapidly.

## Public Health Implications

1. Early appropriate and systematic public health intervention can mitigate projected bad outcomes of an epidemic or pandemic like COVID-19.
2. Partnerships and community-based intervention in an integrative medical fashion that address cultural practices, like congregate living can reduce the impact of public health emergencies
3. An integrative medical approach with early involvement of the decision makers and establishment of a structured control programs helps achieve expected results with minimal morbidities and mortalities.
